# Caspase-8 activation in neutrophils facilitates autoimmune kidney vasculitis through regulating CD4^+^ effector memory T cells

**DOI:** 10.3389/fimmu.2022.1038134

**Published:** 2022-11-25

**Authors:** Jian Hu, Zhen Huang, Min Yu, Pei Zhang, Zhengkun Xia, Chunlin Gao

**Affiliations:** ^1^ Department of Pediatrics, Jinling Hospital, Nanjing Medical University, Nanjing, Jiangsu, China; ^2^ State Key Laboratory of Biotherapy, Key Laboratory of Birth Defects and Related Diseases of Women and Children of MOE, West China Second University Hospital, Sichuan University, Chengdu, China; ^3^ Department of Pediatrics, Affiliated Jinling Hospital, Medical School of Nanjing University, Nanjing, Jiangsu, China

**Keywords:** anti-neutrophil cytoplasmic antibody (ANCA), kidney vasculitis, single-cell transcriptome, neutrophil, caspase-8, immunity

## Abstract

Anti-neutrophil cytoplasmic antibody (ANCA)-associated vasculitides (AAVs) are closely associated with neutrophil recruitment and activation, but the impact of the neutrophil apoptosis process in autoimmune disease has been rarely explained. Here, by integrating and analyzing single-cell transcriptome datasets, we found that the caspase-8-associated pathway in neutrophils was highly activated in the kidney rather than in the blood. To verify the function of caspase-8 in neutrophils on AAVs progression, we constructed neutrophil-specific caspase-8 knockout mice combined with an AAVs model induced by human ANCA from AAVs patients, a rapid and powerful model developed in this study. Our results show that caspase-8 activation of neutrophils up-regulates the expression of several inflammatory and immunoregulatory factors, especially IL23A, regulating the activation and differentiation of tissue-resident CD4^+^ effector memory T cells. This study reveals that the activation of caspase-8 in neutrophils can worsen glomerulonephritis of AAVs by regulating inflammation and immunity.

## Introduction

Anti-neutrophil cytoplasmic antibody (ANCA)-associated vasculitides (AAVs) are frequently life-threatening diseases characterized by inflammation of blood vessels and tissue damage. The most severe form of AAVs is manifested as acute kidney and lung injury or failure (1). Based on typical phenotype, there are three types: granulomatosis with polyangiitis (GPA), microscopic polyangiitis (MPA) and eosinophilic GPA (EGPA).

There are manifold risk factors involved in the development of AAVs, including pathogen infection (2), aging (3), genetic contribution (4), drug reactions (5), and environmental exposure (6), but the mechanisms underlying AAVs have not been well explained. Its physiopathology is based on a loss of tolerance to neutrophil primary granule proteins, most often leukocyte proteinase 3 (PR3) or myeloperoxidase (MPO) (7). Other autoantigens include lysosome-associated membrane protein 2 (LAMP2) (8), complementary PR3 (cPR3) peptides (9), moesin (10), plasminogen (11), peroxidasin (12) and pentraxin 3 (13). There is a consensus that the loss of tolerance to these neutrophil cytoplasmic proteins in T and B cells is an essential step, followed by excessive activation of neutrophils by autoantigen antibodies. Subsequently, activated neutrophils localize on fragile microvascular beds, and neutrophils induce tissue damage while releasing self-antigens presented by antigen-presenting cells such as dendritic cells, allowing effector T cells to recognize the antigen and mediate further damage (14).

In particular, the priming and activation of neutrophils are also critical steps in this process, in which the MPO or PR3 in the cytoplasmic granules will be transferred to the cell membrane surface and then recognized by ANCA (15). Neutrophil priming in AAVs occurs by several mechanisms, of which the most well defined are the complement system (16), Toll-like receptor (TLR) signaling (17), and cytokines (including TNFα and IL-18) (18, 19). Among them, the activation and priming of neutrophils by TNFα in AAVs have been widely reported and applied to clinical therapeutics (20). TNFα is also a crucial factor for inflammation and cell apoptosis by activating the caspase-8 pathway (21). There is interest in determining whether and how the apoptotic status of neutrophils is correlated with the AAVs progression. Meanwhile, single-cell transcriptome sequencing (sc-RNAseq) technology has developed rapidly in recent years, expounding the immune atlas of many organs and diseases (22). In kidney-related diseases, several studies using sc-RNAseq have been recently reported, including diabetic nephropathy (23), IgA nephropathy (24), lupus nephritis (25) and ANCA-associated glomerulonephritis (GN) (26). In this study, we explore the question by combining integrated analysis of sc-RNAseq data and neutrophil-specific caspase-8 knockout mice based on Cre-lox system.

## Materials and methods

### Mice

C57BL/6J and Balb/c mice were purchased from the Model Animal Research Center of Nanjing University. B6.Cg-Tg(S100A8-cre,-EGFP)1Ilw/J (*Cre* ^MRP8^) (27) and B6.129-Casp8tm1Hed/J (*Caspase8* ^LoxP/LoxP^) (28) mice were purchased from The Jackson Laboratory. Eight- to twelve-week-old mice were used for experiments. All mice were housed in specific-pathogen-free conditions and fed with autoclaved food at the Experimental Center of Jinling Hospital, Nanjing Medical University. The protocols of the animal experiments were approved by the Laboratory Animal Ethics Committee of Jinling Hospital, Nanjing Medical University.

### Preparation of human IgG samples

Plasma was collected from healthy donors and patients with active anti-MPO or anti-PR3 positive vasculitis from Jinling Hospital, Nanjing Medical University. Clinical data were collected in a blinded, coded manner. All fulfilled Chapel Hill consensus classification criteria for diagnosis of systemic small vessel vasculitis (29). Total IgG was separated from plasma and tested as detailed previously (30). IgG preparations were added at a concentration of 200 mg/ml.

### Construction of mouse AAVs model

Before the passive transfer of human ANCA to mice, Lipopolysaccharide (LPS) (1500 EU/g/IP) was injected into selected mice for mobilizing and priming neutrophils as described previously (30). Thirty minutes later, mice received an IV injection of 8 mg of human IgG (200 μl). Human IgG from healthy volunteers and AAVs patients were injected under the same conditions. Urine was collected on d1, d3, d6, d10, and d14 and analyzed for hematuria and proteinuria. Mice were sacrificed on the 14th day, and the tissues of each mouse were collected for subsequent analysis, including kidney, lung, spleen, heart, liver, blood, and urine.

### Blood biochemical tests

Serum of mice was used for blood biochemical tests to detect changes in metabolites. The indexes of liver function were: alanine aminotransferase (ALT), total bilirubin (BILT), direct bilirubin (BILD), alkaline phosphatase (ALP), lactate dehydrogenase (LDH), total protein (TP), albumin (ALB), and blood glucose (GLU). The indexes of kidney function were: urea (UREA), creatinine (CREA), uric acid (UA), triglyceride (TG), amylase (AMY), cholesterol (CHOl), low-density lipoprotein cholesterol (LDL-C), and high-density lipoprotein Cholesterol (HDL-C).

### Immunofluorescence and pathological analysis of organs

The fresh tissues were embedded with opti-mum cutting temperature compound (O.C.T. Compound), frozen at -80°C, and cut into sections for immunofluorescence on a freezing microtome (Leica). Then, sections were incubated in blocking solution (0.4% Triton X-100 and 10% donkey serum in phosphate buffer saline) for 30 min at room temperature, followed by incubating in blocking solution containing primary antibodies overnight at 4 °C in the dark. Primary antibodies used were mouse anti-MPO (1:200, 66177-1-Ig, Proteintech), rabbit anti-IL-23 antibody (1:50, bs-1193R, Bioss), and rat anti-CD4 (1:200, ab34276, abcam). Sections were washed in wash buffer three times and then incubated in a blocking solution containing secondary antibodies for two hours at room temperature, followed by incubating in 4’,6-diamidino-2-phenylindole (DAPI) (1:5,000, 10236276001, Roche) for 15 min. As previously described (31), the presence of circulating anti-MPO antibodies was confirmed in selected animals by indirect immunofluorescence microscopy assay on mouse neutrophils. Mouse neutrophils from bone marrow were incubated with serum from groups, followed by an incubation with donkey antibodies against mouse IgG. Images of stained slices were acquired using a confocal microscope (LSM980, Carl Zeiss). The paraffin sections of the heart, liver, lung and kidney were stained with PAS or H&E and analyzed for pathological changes.

### Quantitative real-time PCR and western blotting

RNA was isolated and reverse-transcribed to cDNA with oligos using the Takara reverse transcriptase system (RR047A), then analyzed using an SYBR Green master mix (E166, novoprotein). A table of human and mouse primers used in this study is shown in **Table S1**.

For western blotting, cells were lysed in 1× RIPA buffer (20-188, millipore) and processed by standard western blot techniques. Membranes were blocked with 5% BSA in phosphate buffer saline (PBS) containing 0.5% Tween-20 and incubated with primary antibodies for β-actin (1:1000, M1210-2, Huabio), caspase-8 (1:1000, 66093-1-Ig, Proteintech), caspase-9 (1:1000, 10380-1-AP, Proteintech), IL-23A (1:200, bs-1193R, Bioss).

### Isolation of neutrophils

Mouse neutrophils from the bone marrow of *Cre* ^MRP8^ *Caspase8* ^LoxP/LoxP^ or *Caspase8* ^LoxP/LoxP^ mice and human neutrophils from peripheral blood of AAVs patients or healthy donors were isolated by density gradient centrifugation, as described previously (19). The isolated neutrophils of mice were centrifuged onto glass slides for indirect immunofluorescence. Most collected neutrophils were used for RT-qPCR and western blotting.

### Integration and analysis of single-cell RNA-seq data

The “Seurat” package was used to perform the single-cell RNA-seq analysis and integration (32). The non-linear dimensional reduction was performed with the UMAP method. Cluster biomarkers were found by the “Seurat” and “SingleR” packages (33). The immunocyte subset was extracted from each dataset and integrated to perform the downstream analysis. The “Seurat” package removed the batch effect from studies. The enrichment scores of the differential expressed genes were evaluated using GSEA software (version: 4.1.0) (34, 35). The KEGG and GO analyses were done in DAVID (36). The cell-cell communication was analyzed by CellPhoneDB (37, 38). The similarity score was analyzed as described previously (39).

### Statistical analysis

Data analyses were done using GraphPad Prism version 8. An unpaired t-test was used to compare the two groups. Multiple t-tests were performed to compare several cell populations or genes between two groups. R software (version: 4.1.0) was used for scRNA-seq analysis. Statistical tests used are indicated in figure legends. All data are shown as means ± s.e.m. *n* values represent biological replicates. Differences were considered significant when p < 0.05 and are indicated as *p < 0.05, **p < 0.01, ***p < 0.001, ****p < 0.0001, ns, not significant.

## Results

### Human ANCA induces systemic AAVs in mice, especially glomerulonephritis

**Figure 1:**
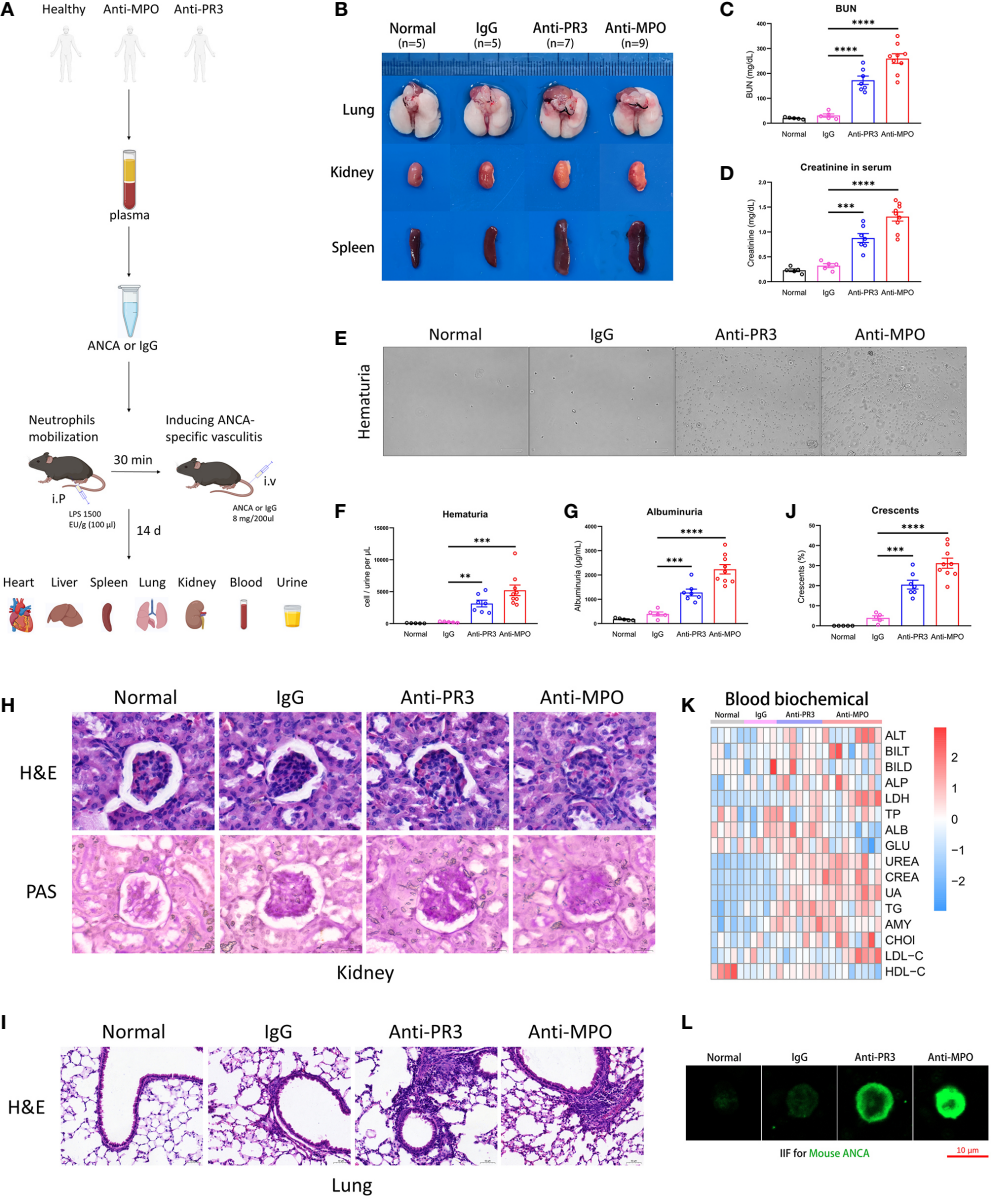
The ANCA-associated vasculitis model of mouse induced by human ANCA from patients. **(A)** The mouse model of ANCA-associated vasculitis, constructed with human IgG from healthy donors, anti-PR3 or anti-MPO vasculitis patients after neutrophil mobilization by LPS. **(B)** The representative pictures of the lung, kidney and spleen from all groups. **(C, D)** The comparison of urea nitrogen **(C)** and creatinine **(D)** in mouse serum. **(E)** The representative pictures of urine smear across all groups. **(F, G)** The comparison of hematuria **(F)** and albuminuria **(G)**. **(H, I)** H&E and PAS staining images of the kidney **(H)** and H&E staining images of the lung **(I)** from mice of all groups. Scale bar, 20 μm. **(J)** The summarized number of crescents among all groups. **(K)** The detection of biochemical indexes in mouse serum. **(L)** The indirect immunofluorescence microscopy assay for mouse ANCA on mouse neutrophils. Scale bar, 10 μm. **P < 0.01, ***P < 0.001, ****P < 0.0001.

First, we sought to construct a fast and intense mouse model of AAVs. We used ANCA from PR3-ANCA and MPO-ANCA patients to directly induce AAVs in mice (**Figure 1A**). Organ phenotypes of the anti-PR3 and anti-MPO groups were obviously different compared to the normal and control IgG groups (**Figure 1B**), mainly in the kidney, lung, and spleen. Inflammatory necrosis was observed in the kidneys of ANCA groups. Inflammatory plaques were present in the lungs of anti-PR3 and anti-MPO groups. The spleens of ANCA groups displayed obvious swelling, indicating a more intense immune response. Besides, our results showed significant up-regulation of serum urea nitrogen and creatinine in both anti-PR3 and anti-MPO groups relative to normal and control IgG groups (**Figure 1C, D**). In addition, immune cells, endothelial cells, and epithelial cells from damaged glomeruli appeared in the urine of ANCA groups, especially anti-MPO-induced mice (**Figure 1E, F**). Subsequently, we found that the levels of albuminuria were higher in mice treated with ANCA (**Figure 1G**). These results indicate that mice developed severe inflammatory and immune responses after human ANCA injection, resulting in tissue damage and failure.

Next, we performed the H&E and PAS staining to observe the pathological changes of tissue damage in mice. Our results showed that the kidneys of ANCA groups had obvious pathological glomerular changes, including the appearance of crescents and necrotic foci, a phenomenon that was almost absent in normal and control IgG mice. Meanwhile, the accumulation of inflammatory cells and tissue remodeling appeared in lung sections of anti-PR3 and anti-MPO mice rather than in normal and control IgG mice (**Figure 1H–J**). Besides, the mice of ANCA groups also developed mild inflammatory responses and pathological changes in the heart and liver (**Supplementary Figure 1A, B**).

To systematically explore the physiological and metabolic changes among different groups, we detected the contents of biochemical blood indexes in four groups (**Figure 1K**). The result showed that kidney function indexes (UREA, CREA, UA, TG, AMY, CHOl, LDL-C, HDL-C) in blood tests were significantly increased in ANCA groups, while liver function indexes (ALT, BILT, BILD, ALP, LDH, TP, ALB, GLU) did not display a consistent trend. In conclusion, these results showed that severe inflammation and kidney impairment occurred in the anti-MPO-treated mice.

We employed indirect immunofluorescence staining to verify the existence of mouse ANCA in the model. The images showed that mouse ANCA was combined with neutrophil cytoplasmic proteins (green), which was present in serum from ANCA groups but absent in normal and control groups (**Figure 1L**). Taken together, these results suggested that the mice treated with human ANCA developed systemic AAVs, especially glomerulonephritis.

### Integration and analysis of the single-cell transcriptome of blood and kidney immunocytes

The role of neutrophils was indispensable in the pathogenesis of AAV-associated kidney disease. To examine heterogeneity of neutrophils in different microenvironment, we selected and integrated immunocytes of human sc-RNAseq datasets (40, 41) containing neutrophils by Seurat (32). First, we extracted immune cell populations, including T cells, B cells, NK cells, and Myeloid cells (**Supplementary Figure 2A, B**). Then, we performed an integrated analysis of the immune cells from two different tissues by Seurat (42).

**Figure 2:**
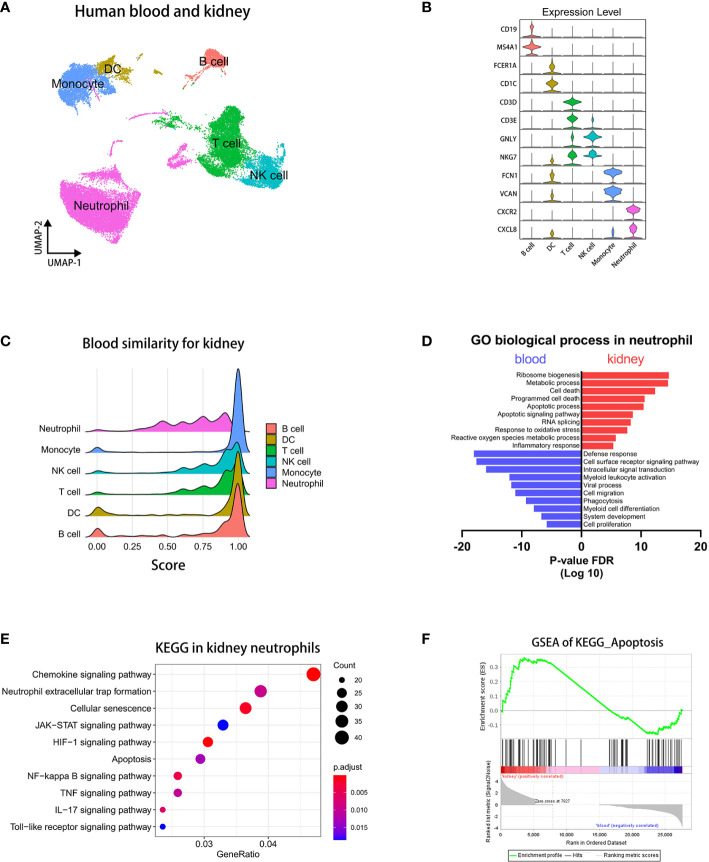
Integrated analysis of sc-RNAseq data reveals pro-apoptotic status in kidney neutrophils. **(A)** UMAP plot clustering of immunocytes in blood and kidney. **(B)** Violin plots displaying the marker genes of identified subsets. **(C)** The bridge plots showing similarity scores in blood subsets comparing to corresponding subsets in the kidney. **(D)** The enrichment analysis of GO biological process in blood and kidney neutrophils. **(E)** The KEGG analysis of signaling pathways in differentially expressed genes of kidney neutrophils. **(F)** The GSEA plots of the apoptosis pathway.

Through the singleR package (33) and the marker genes previously reported (43), we performed a UMAP visualization and annotated all subsets (**Figure 2A**). The violin and heatmap plots were used to display the most significant gene signatures expressed in each immune subset as follows (**Figure 2B** and **Supplementary Figure 2C**): B cells (*CD19*, *MS4A1*), DC (*FCER1A*, *CD1C*), T cells (*CD3D*, *CD3E*), NK cells (*GNLY*, *NKG7*), monocytes (*FCN1*, *VCAN*), and neutrophils (*CXCR2*, *CXCL8*).

### Kidney neutrophils significantly up-regulated caspase-8 pathways correlated with AAVs

To explore difference of immunocytes between blood and kidney, we performed a corresponding similarity analysis of six immunocyte subsets, as previously described (39). A bridge plot was used to visualize similarity scores, which indicated the similarity of immune cells in blood relative to the kidney (**Figure 2C**). The result indicates that neutrophils have more heterogeneities than other immunocytes between blood and kidney. We then colored the differential scores of each immune cell in the UMAP plot according to the similarity scores above, in which differences of neutrophils between blood and kidney were distinctly marked (**Supplementary Figure 3A**).

Then, we employed the volcano plot to visualize differentially expressed genes of neutrophils between blood and kidney (**Supplementary Figure 3B**). We found that kidney neutrophils significantly up-regulated more genes, most of which were associated with metabolism, ATP synthesis, and the ribosome. It indicated that neutrophils were more active in the renal microenvironment and required more energy supply and protein synthesis. And blood neutrophils significantly up-regulated *MT-RNR2* (negative regulation of cell death) (44), *MT-RNR1* (regulation of carbohydrate utilization and phosphate metabolic process) (45), and *IGKC* (predicted to enable antigen and immunoglobulin receptor binding activity) (46) genes. To compare the two types of neutrophils in status and function, we performed an enrichment analysis of the GO biological process on the differentially expressed genes (36). The results showed that the genes of kidney neutrophils were highly enriched in apoptosis-related processes in addition to ribosome- and metabolism-related processes, while the genes of blood neutrophils were more abundant in defense response, cell surface receptor signaling pathway, and intracellular signal transduction (**Figure 2D**). The KEGG analysis (36) similarly showed that apoptosis-associated pathways were significantly increased such as cellular senescence, apoptosis, NF-kappa B signaling pathway, and TNF signaling pathway (**Figure 2E**). In particular, neutrophil extracellular trap formation (NETosis), a specific death of neutrophils contributing to the development of AAVs (47), was also enriched in differentially expressed genes of kidney neutrophils. The GSEA analysis (34, 35) showed that kidney neutrophils significantly up-regulated apoptosis-related genes overall (**Figure 2F**).

**Figure 3:**
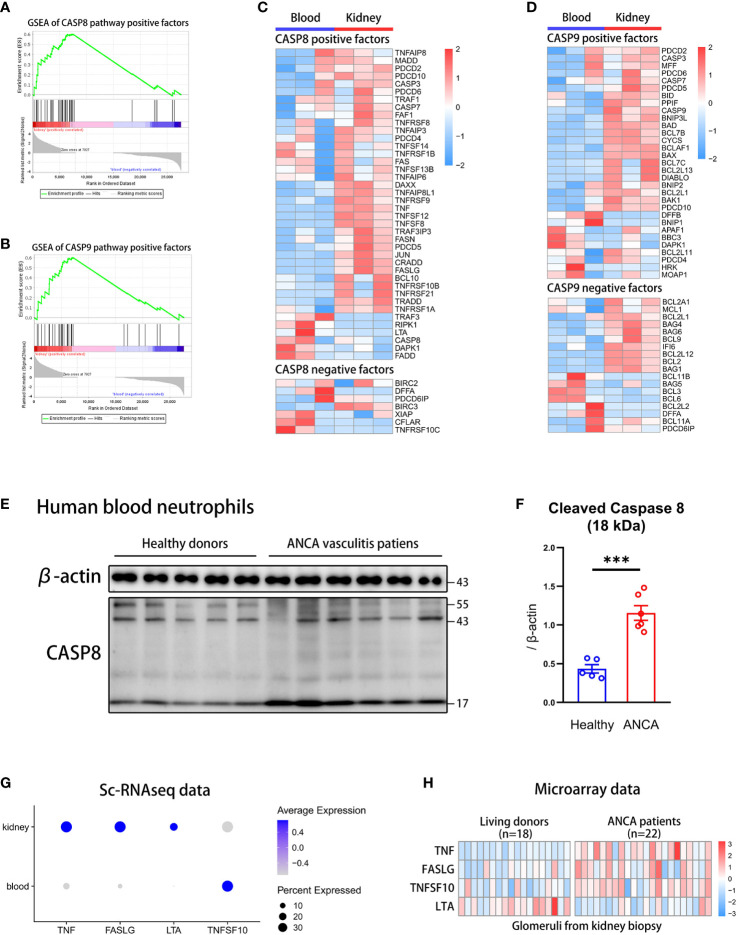
The caspase-8 activation in neutrophils is highly correlated with ANCA-associated vasculitis. **(A, B)** The GSEA plots of positive regulation genes for caspase-8 **(A)** or caspase-9 **(B)** pathway in kidney neutrophils. **(C, D)** The heatmap displaying gene expression for caspase-8 **(C)** or caspase-9 **(D)** pathway regulation in blood and kidney neutrophils. **(E)** Western blot of caspase-8 in human blood neutrophils from healthy donors and ANCA-associated vasculitis patients. **(F)** The summary plot comparing levels of cleaved caspase-8 (18 kDa) relative to β-actin between healthy and ANCA groups. **(G)** The dot plot showing the expression of exogenous positive factors for the caspase-8 pathway in blood and kidney tissues through sc-RNAseq data. **(H)** The heatmap comparing the expression of exogenous positive factors for the caspase-8 pathway in glomerulis between living donors and ANCA patients through microarray data. ***P < 0.001.

To know which apoptosis pathway is raised in kidney neutrophils, we performed a GSEA analysis of positive regulator genes on the exogenous caspase-8 pathway and endogenous caspase-9 pathway. The caspase-8 and caspase-9 pathway-positive factors were significantly increased in neutrophils from the kidney but not from the blood (**Figure 3A, B**). The heatmap showed that most of the positive regulation genes of the caspase-8 pathway were significantly up-regulated in kidney neutrophils, while negative regulation genes were down-regulated overall (**Figure 3C**). For the caspase-9 pathway, although positive regulation genes were raised, many negative regulation genes were significantly up-regulated in kidney neutrophils (**Figure 3D**).

Although we found that apoptosis pathways of kidney neutrophils were highly activated, whether the apoptotic phenotype was correlated with the occurrence of AAVs was unclear. To verify this question, we extracted blood neutrophils from AAVs patients and healthy donors for western blot analysis of caspase-8 and caspase-9. We found that caspase-8 was significantly activated and sheared in neutrophils from AAVs patients, especially the 18 kDa spliceosome (**Figure 3E, F**) (48), and the 35 kDa spliceosome of caspase-9 was slightly increased in neutrophils from AAVs patients (**Supplementary Figure 4A, B**). The above results indicate that the caspase-8 activation in neutrophils was closely related to the development of human AAVs.

However, the activation of the caspase-8 pathway was mainly regulated by exogenous stimulatory factors, such as TNFα, FASL, LTA, and TNFSF10 (49). To further detect the expression of stimulators in the kidney microenvironment, we analyzed sc-RNAseq and microarray data. The dot plot showed that *TNF*, *FASLG*, and *LTA* were significantly raised in the kidney but not *TNFSF10* (**Figure 3G**). We then analyzed the microarray data of glomerulis from AAVs patients (n=22) and healthy donors (n=18) (50). The heatmap plot showed that glomerulis from AAVs patients had more expression of *TNF*, *FASLG*, and *TNFSF10* but not *LTA* (**Figure 3H**). Taken together, our results suggest a significant positive correlation between caspase-8 activation of neutrophils and the development of human AAVs.

### Knockout of caspase-8 in neutrophils significantly alleviates AAVs phenotype in mice

**Figure 4:**
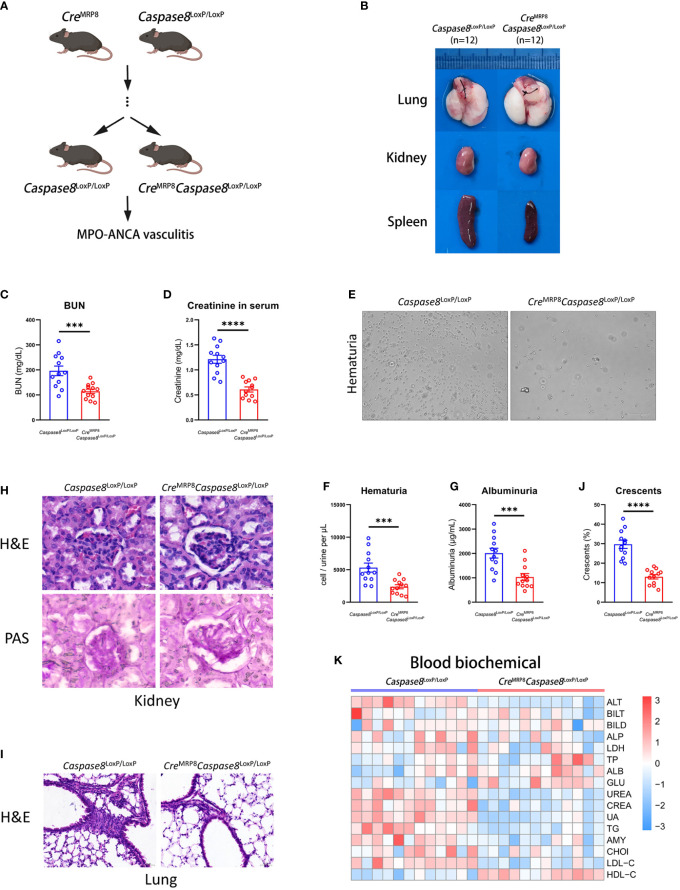
The caspase-8 knockout in neutrophils alleviates systemic vasculitis in mice. **(A)** Breeding strategies of *Cre* ^MRP8^ *Caspase8* ^LoxP/LoxP^ mice. **(B)** The representative pictures of lung, kidney and spleen from *Caspase8* ^LoxP/LoxP^ and *Cre* ^MRP8^ *Caspase8* ^LoxP/LoxP^ mice. **(C, D)** The comparison of urea nitrogen **(C)** and creatinine **(D)** in mouse serum between two groups. **(E)** The representative pictures of urine smear from groups. **(F, G)** The comparison of hematuria **(F)** and albuminuria **(G)** between *Caspase8* ^LoxP/LoxP^ and *Cre* ^MRP8^ *Caspase8* ^LoxP/LoxP^ mice. **(H, I)** H&E and PAS staining images of the kidney **(H)** and H&E staining images of the lung **(I)** from two groups. Scale bar, 20 μm. **(J)** The summarized number of crescents according to H&E images. **(K)** The detection of biochemical criteria in mouse serum from *Caspase8* ^LoxP/LoxP^ and *Cre* ^MRP8^ *Caspase8* ^LoxP/LoxP^ mice. ***P < 0.001, ****P < 0.0001.

To explore the effect of caspase-8 in neutrophils on the development of AAVs, we constructed the *Cre* ^MRP8^ *Caspase8* ^LoxP/LoxP^ mice (27, 28) for the caspase-8 knockout in neutrophils based on the Cre-lox system (**Figure 4A**). The *Caspase8* ^LoxP/LoxP^ mice were used as the control group. For genotyping, *Cre* ^MRP8^ *Caspase8* ^LoxP/LoxP^ and *Caspase8* ^LoxP/LoxP^ mice were screened out by nucleic acid electrophoresis with corresponding primers (**Supplementary Figure 5A**). And the results showed that the neutrophils from *Cre* ^MRP8^ *Caspase8* ^LoxP/LoxP^ mice had lower expression of caspase-8 in transcript and protein levels (**Supplementary Figure 5B**).

After being injected with human MPO-ANCA for 2 weeks, there were significant differences in the kidney, lung, and spleen between *Cre* ^MRP8^ *Caspase8* ^LoxP/LoxP^ (n=12) and *Caspase8* ^LoxP/LoxP^ (n=12) mice (**Figure 4B**). It could be seen from the representative pictures that the inflammation in the kidney, lung, and spleen was lower in *Cre* ^MRP8^ *Caspase8* ^LoxP/LoxP^ mice. In addition, urea nitrogen, creatinine, hematuria, and proteinuria of *Cre* ^MRP8^ *Caspase8* ^LoxP/LoxP^ mice were lower compared to *Caspase8* ^LoxP/LoxP^ mice (**Figure 4C–G**). These results showed that kidney function failure caused by AAVs may be at least partially prevented by caspase-8 knockout in neutrophils.

We then performed histopathological analysis to visually compare the phenotypic differences between the two groups. The results of H&E and PAS staining in mouse kidneys showed that a lower grade and proportion of glomerular crescents were present in kidneys from *Cre* ^MRP8^ *Caspase8* ^LoxP/LoxP^ mice. The inflammation and necrosis in lung, heart, and liver of the knockout mice were significantly reduced. Finally, we detected the biochemical indexes in the serum of knockout and control mice, and the results displayed in the heatmap showed that kidney-related indexes were significantly reduced (**Figures 4H–J**; **Supplementary Figures 5C, D**) in *Cre* ^MRP8^ *Caspase8* ^LoxP/LoxP^ mice (**Figure 4K**). These results indicate that the knockout of caspase-8 in neutrophils effectively alleviated the phenotype caused by AAVs in mice.

### Neutrophils strongly interact with effector memory CD4^+^ T cells in kidney microenvironment

**Figure 5:**
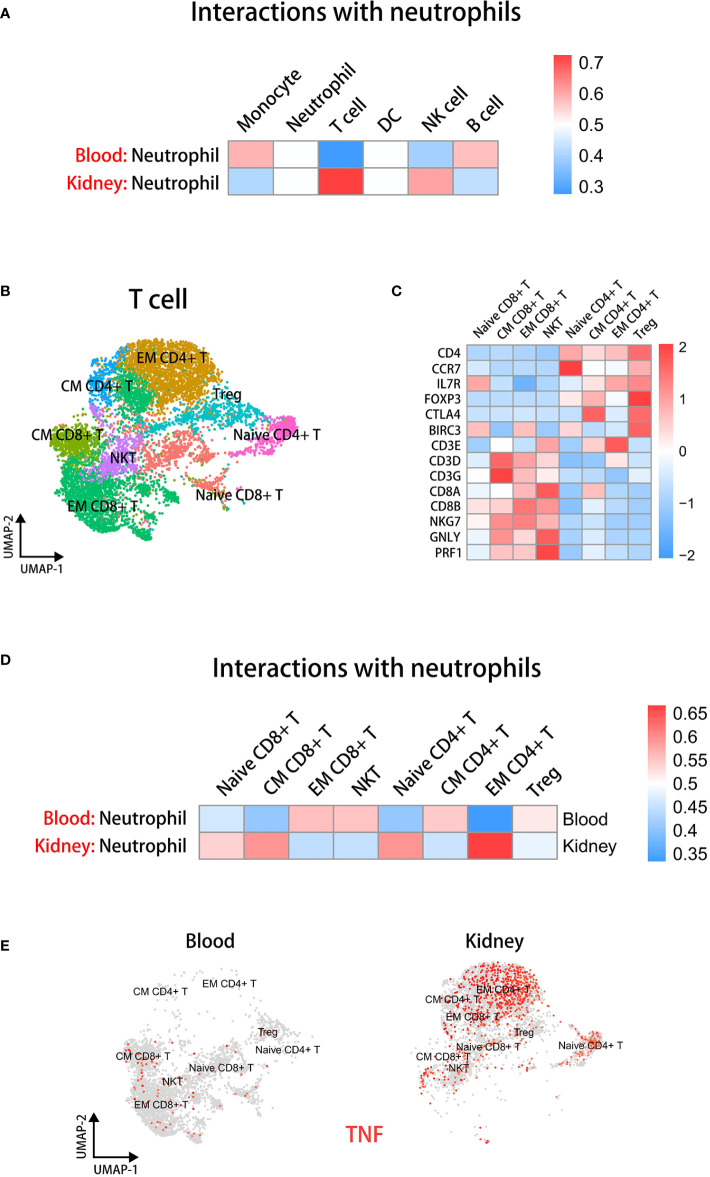
Neutrophils have significant interactions with EM CD4+ T cells in the kidney microenvironment. **(A)** The cell-cell communication analysis displaying interactions between neutrophils and other immune cells in blood or kidney datasets. **(B)** The UMAP plot of T cell subsets in blood and kidney datasets. **(C)** The heatmap showing the expression of marker genes in identified subsets. **(D)** The cell-cell communication analysis displaying interactions between neutrophils and T cell subsets in blood or kidney datasets. **(E)** The UMAP plot colored by TNF in T cell subsets in blood and kidney groups.

To compare the differences in communications of neutrophils and other immune cells between the blood and kidney environment, we employed the CellphoneDB (37, 38) to analyze the interactions among immunocytes in blood and kidney respectively from sc-RNAseq data. The results showed that the interactions between neutrophils and T cells were most significantly increased in the kidney relative to blood (**Figure 5A**).

To further elucidate which kinds of T cells interacted with kidney neutrophils, we extracted T cells individually from integrated sc-RNAseq data. Then we performed dimensionality reduction again and used Clustree analysis (51) to select an appropriate resolution for clustering (**Supplementary Figure 6A**). T cells were classified into eight subsets according to marker genes as previously described (43), including Naive CD4+ T, CM CD4+ T, EM CD4+ T, Treg, Naive CD8+ T, CM CD8+ T, EM CD8+ T, and NKT (**Figure 5B**). A heatmap displays marker gene expression of T cell subsets (**Figure 5C**). We found that the EM CD4+ T cells closely communicated with neutrophils in the kidney environment (**Figure 5D**).

Next, we performed UMAP visualization colored by *TNF* and *FASL* across tissues to examine which kinds of T cell subsets could stimulate the caspase-8 pathway of neutrophils (**Figure 5E** and **Supplementary Figure 6B**). The results indicated that a higher proportion of EM CD4+ T cells were present in the kidney and *TNF* but not *FASLG* expression was significantly up-regulated in EM CD4+ T cells of kidney.

### A variety of inflammatory and immunoregulatory factors are produced through the activation of caspase-8 in neutrophils, especially IL23A

**Figure 6:**
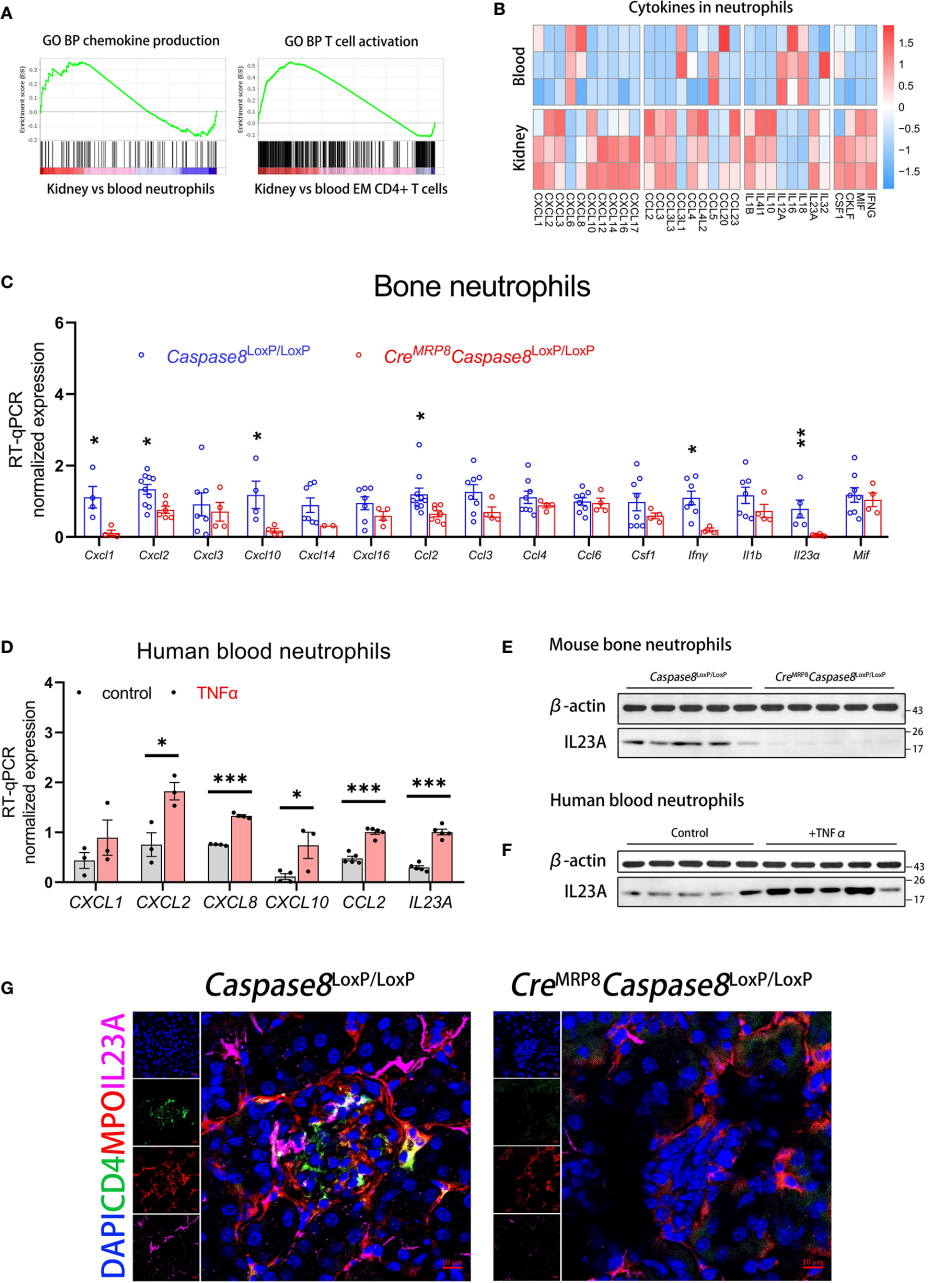
Neutrophils can produce IL23A for regulating CD4^+^ memory T cells through caspase-8 activation. **(A)** The GSEA plots of chemokine production in neutrophils and T cell activation in EM CD4+ T cells for kidney versus blood. **(B)** The heatmap comparing the expression of inflammation and immunoregulation factors between blood and kidney neutrophils. **(C)** RT-qPCR analysis of bone neutrophils for genes significantly up-regulated in kidney neutrophils between *Caspase8* ^LoxP/LoxP^ and *Cre* ^MRP8^ *Caspase8* ^LoxP/LoxP^ mice. **(D)** RT-qPCR analysis of human blood neutrophils detecting gene expression changes after TNFα stimulation *in vitro*. **(E)** The western blot showing protein expression of IL23A in bone neutrophils from *Caspase8* ^LoxP/LoxP^ and *Cre* ^MRP8^ *Caspase8* ^LoxP/LoxP^ mice. **(F)** The western blotting showing protein expression of IL23A in human blood neutrophils between control and TNFα groups. **(G)** The immunofluorescence shoots showing the relationship between IL23A (pink) secreted by neutrophils (red) and CD4^+^ T cells (green) in the kidney microenvironment of *Caspase8* ^LoxP/LoxP^ and *Cre* ^MRP8^ *Caspase8* ^LoxP/LoxP^ mice. Scale bar, 20 μm. *P < 0.05, **P < 0.01, ***P < 0.001.

To explore the mechanism by which neutrophils promoted AAVs through regulating inflammation and immunity, we performed a GSEA analysis of neutrophils and EM CD4+ T cells in kidney relative to blood. We found that kidney neutrophils had a greater capacity for chemokine production than blood neutrophils, and there was a higher degree of T cell activation in kidney EM CD4+ T cells than in blood (**Figure 6A**). We analyzed the differentially expreesed expression of related cytokines between blood and kidney neutrophils to determine the factors contributing to inflammation and immunomodulatory for AAVs development, as shown in heatmap plots (**Figure 6B**).

Next, we selected factors greatly up-regulated in kidney neutrophils to compare their expression levels in bone neutrophils from *Cre* ^MRP8^ *Caspase8* ^LoxP/LoxP^ and *Caspase8* ^LoxP/LoxP^ mice. RT-qPCR analysis showed that *Cxcl1*, *Cxcl2*, *Cxcl10*, *Ccl2*, *Ifnγ*, and *Il23a* expression in neutrophils were significantly down-regulated in *Cre* ^MRP8^ *Caspase8* ^LoxP/LoxP^ mice (**Figure 6C**). The transcriptional expression of *Il1b*, reported being associated with AAVs (52), had non statistically difference but a downward trend in neutrophils from knockout mice. To verify whether these factors were also regulated by the activation of caspase-8 in human neutrophils, we stimulated blood neutrophils from healthy donors with TNFα *in vitro*, and we found that *CXCL2*, *CXCL8*, *CXCL10*, *CCL2*, and *IL23A* expression were also up-regulated in human neutrophils (**Figure 6D**).

Among these factors, *IL23A* was more significantly regulated in both mouse and human neutrophils. As IL23A associates with IL12B to form the cytokine IL-23 reported contributing to the development of AAVs (14, 53, 54), we examined the protein expression levels of IL23A in mouse bone neutrophils and human blood neutrophils. Western blotting showed that the protein expression of IL23A was lower in *Cre* ^MRP8^ *Caspase8* ^LoxP/LoxP^ compared to *Caspase8* ^LoxP/LoxP^ group (**Figure 6E**) and was significantly up-regulated after TNFα activated caspase-8 in human blood neutrophils (**Figure 6F**). Finally, to more intuitively observe the correlation and co-localization of IL23A, neutrophils, and CD4^+^ T cells in the kidney microenvironment, we performed immunofluorescent staining in kidney sections of *Cre* ^MRP8^ *Caspase8* ^LoxP/LoxP^ and *Caspase8* ^LoxP/LoxP^ mice developing AAVs. The images indicated that neutrophils (MPO, red) and CD4^+^ T cells (CD4, green) aggregated together in the necrotic glomeruli of *Caspase8* ^LoxP/LoxP^ mice, followed by IL23A (pink) abundantly enriched around the cells. In contrast, there were fewer neutrophils, CD4+ T cells in restored glomeruli after caspase-8 was knocked out in neutrophils, along with less IL23A deposition locally (**Figure 6G**).

### The IL23-mediated pathway was greatly activated in EM CD4+ T cells from glomeruli of AAVs patients

**Figure 7:**
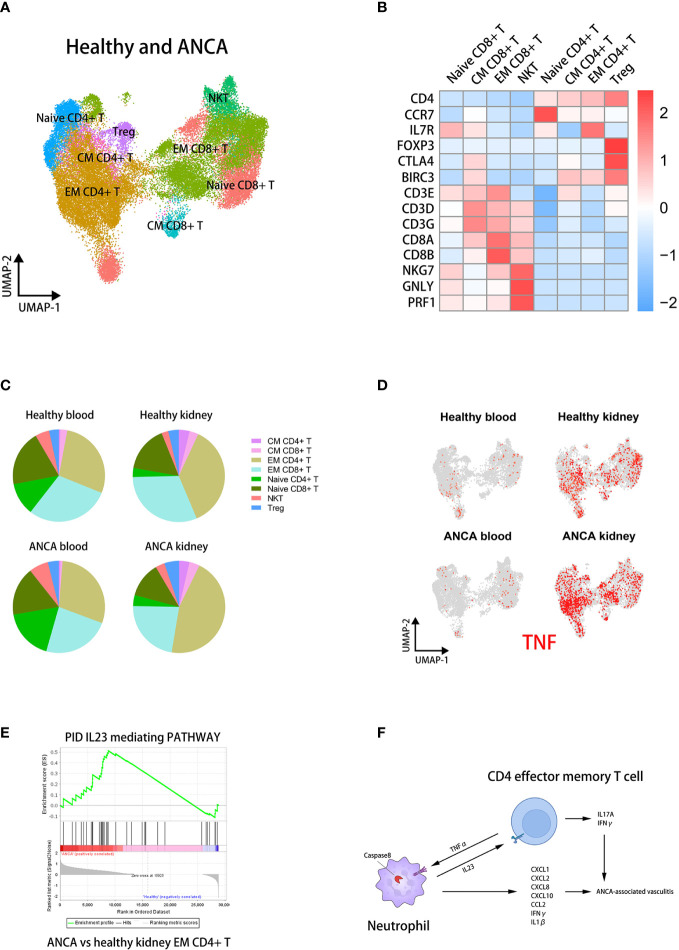
The IL23 mediating pathway in kidney EM CD4+ T cells is positively correlated with AAVs. **(A)** UMAP clustering of healthy and ANCA T cells in human blood and kidney sc-RNAseq datasets. **(B)** The heatmap showing the expression of marker genes in identified subsets. **(C)** The pie plots comparing the proportion of T cell subsets across groups. **(D)** The UMAP plot colored by TNF in T cell subsets of four groups. **(E)** The GSEA plots of the IL23-mediating pathway in EM CD4+ T cells for ANCA versus healthy kidney. **(F)** The cross-talk between neutrophils and CD4+ effector memory T cells contributing to the development of ANCA-associated vasculitis.

To verify whether the ratios and IL23 stimulation of EM CD4+ T cells were closely correlated with AAVs development, we integrated sc-RNAseq datasets of T cells from healthy donors and AAVs patients previously reported (26, 40, 41). The appropriate resolution was selected by Clustree for dimensionality reduction (**Supplementary Figure 7A**). All cells were annotated by group labels in the UMAP plot (**Supplementary Figure 7B**). Similarly, according to the previously reported marker genes (43), we defined the subsets as Naive CD4+ T, CM CD4+ T, EM CD4+ T, Treg, Naive CD8+ T, CM CD8+ T, EM CD8+ T, and NKT (**Figure 7A**). A heatmap showed marker gene expression across T cell subsets (**Figure 7B**).

We then found that the proportion of EM CD4+ T cells was significantly increased in the kidney of AAVs patients (**Figure 7C**). We analyzed the differential expression of *TNF* in each group and T cell subset, shown in UMAP plots (**Figure 7D**). It can be seen that *TNF* was specifically up-regulated in the kidney of AAVs patients compared with other groups. In particular, *TNF* was mainly expressed in EM CD4+ T cells of kidney, similar to the above results. This suggested that T cells, mainly EM CD4+ T cells, could stimulate the caspase-8 activation of neutrophils through TNFα in the kidney environment of AAVs patients. Furthermore, GSEA analysis suggested a stronger IL23 stimulation of EM CD4+ T cells in the kidney environment of AAVs (**Figure 7E**). In conclusion, the positive feedback between neutrophils and EM CD4+ T cells ultimately promotes the development of glomerulitis caused by ANCA (**Figure 7F**).

## Discussion

Our study revealed that the activation of caspase-8 in neutrophils can facilitate the development of ANCA-associated vasculitis through the regulation of inflammation and immune response. Despite inflammatory factors, neutrophils can produce immune regulator IL23 to promote the activation and differentiation of kidney-resident EM CD4+ T cells after activation of caspase-8. We describe a mouse model of AAVs using human ANCA, which is different from the models of active or passive immunization by using mouse antigens or antibodies against mouse antigens as previously reported (55). The model used in this study facilitates mechanistic research of AAVs.

The critical role of TNFα in ANCA-associated vasculitis have been widely reported, mainly as the function of priming and activating neutrophils (15). Meanwhile, as a typical apoptosis stimulator, the TNFα could also accelerate the apoptosis process of neutrophils and activate apoptosis-related inflammation (21). Although neutrophil apoptosis was closely correlated with inflammation, there was no evidence that neutrophil apoptosis status was directly related to the development of AAVs. In our study, we employed neutrophil-specific caspase-8 knockout mice to demonstrate that the knockout of caspase-8 in neutrophils effectively decreased the progression of AAVs in mice, especially glomerulitis. We also found that the activation of caspase-8 in neutrophils produced several inflammatory chemokines to directly participate in the kidney inflammatory process, and up-regulated immunoregulation factors to indirectly exacerbate the local vasculitis through the activation of CD4^+^ effector memory T cells.

Tissue-resident memory T cells have been reported as a crucial role in the development of AAVs in numerous studies, including Th1, Th2, and Th17 cells (56). After activation by IL-23, the memory CD4^+^ T cells are differentiated into T_H_17 cells, which highly express IFNγ, TNFα, and IL17A, resulting in the excessive activation of local neutrophils and tissue damage (57). We found that activation of caspase-8 in neutrophils significantly up-regulated the expression of IL23A, whether by transcript or protein levels. It was previously reported that IL23A combined with IL12B could stimulate the activation and differentiation of CD4^+^ memory T cells (53, 54). Nevertheless, IL23A was derived from antigen-presenting cells such as DCs or macrophages in most cases, but our study found a new source of IL23A. This indicated that caspase-8-dependent neutrophil activation could regulate local CD4^+^ effector memory T cells to promote the development of glomerular vasculitis in AAVs. In turn, the activated CD4^+^ effector memory T cells can also overexpress TNFα in kidney of AAVs, which activates and primes local neutrophils.

The cross-talk between neutrophils and CD4^+^ effector memory T cells revealed in this study suggests that the development of AAVs nephritis is a process of interactions between various immune cells, in which activation of neutrophils can act on T cells through the production of immunoregulatory factors and the activated T cells can also secrete cytokines to excessively activate neutrophils in turn. The feedback process in this study can provide new insights for multi-target therapeutic strategies of AAVs.

## Data availability statement

Publicly available datasets analyzed in this study can be found online repositories. The names of the repository/repositories and accession number(s) can be found below: https://www.ncbi.nlm.nih.gov/geo/query/acc.cgi?acc=GSE190329, GSE190329, GEO, NCBI; https://www.ncbi.nlm.nih.gov/geo/query/acc.cgi?acc=GSE104948, GSE104948, GEO, NCBI; https://data.humancellatlas.org/explore/projects/abe1a013-af7a-45ed-8c26-f3793c24a1f4, ERP120466, Human Cell Atlas; https://figshare.com/s/7912de1afc7fd5bbefd4, figshare.

## Ethics statement

The animal study was reviewed and approved by the Laboratory Animal Ethics Committee of Jinling Hospital, Nanjing Medical University.

## Author contributions

JH and ZH designed and performed the experiments, analyzed the data, and wrote the manuscript. JH and ZH performed the experiments. MY and PZ provided suggestions on the project design. ZX and CG conceived the project, designed and supervised the experiments, and revised the manuscript. All authors contributed to the article and approved the submitted version.

## Funding

This study was supported by the Natural Science Foundation & Youth Fund Plan of Jiangsu Province (BK20190251); China Postdoctoral Foundation (2018M643888); Postdoctoral Fund of Jiangsu Province (2018K089B).

## Conflict of interest

The authors declare that the research was conducted in the absence of any commercial or financial relationships that could be construed as a potential conflict of interest.

## Publisher’s note

All claims expressed in this article are solely those of the authors and do not necessarily represent those of their affiliated organizations, or those of the publisher, the editors and the reviewers. Any product that may be evaluated in this article, or claim that may be made by its manufacturer, is not guaranteed or endorsed by the publisher.
