# Supplementary figures and images for: Caspase-8 activation in neutrophils facilitates autoimmune kidney vasculitis through regulating CD4+ effector memory T cells
Source: Front Immunol. 2022 Nov 25;13:1038134. doi: 10.3389/fimmu.2022.1038134 (PMC9732547; doi:10.3389/fimmu.2022.1038134)

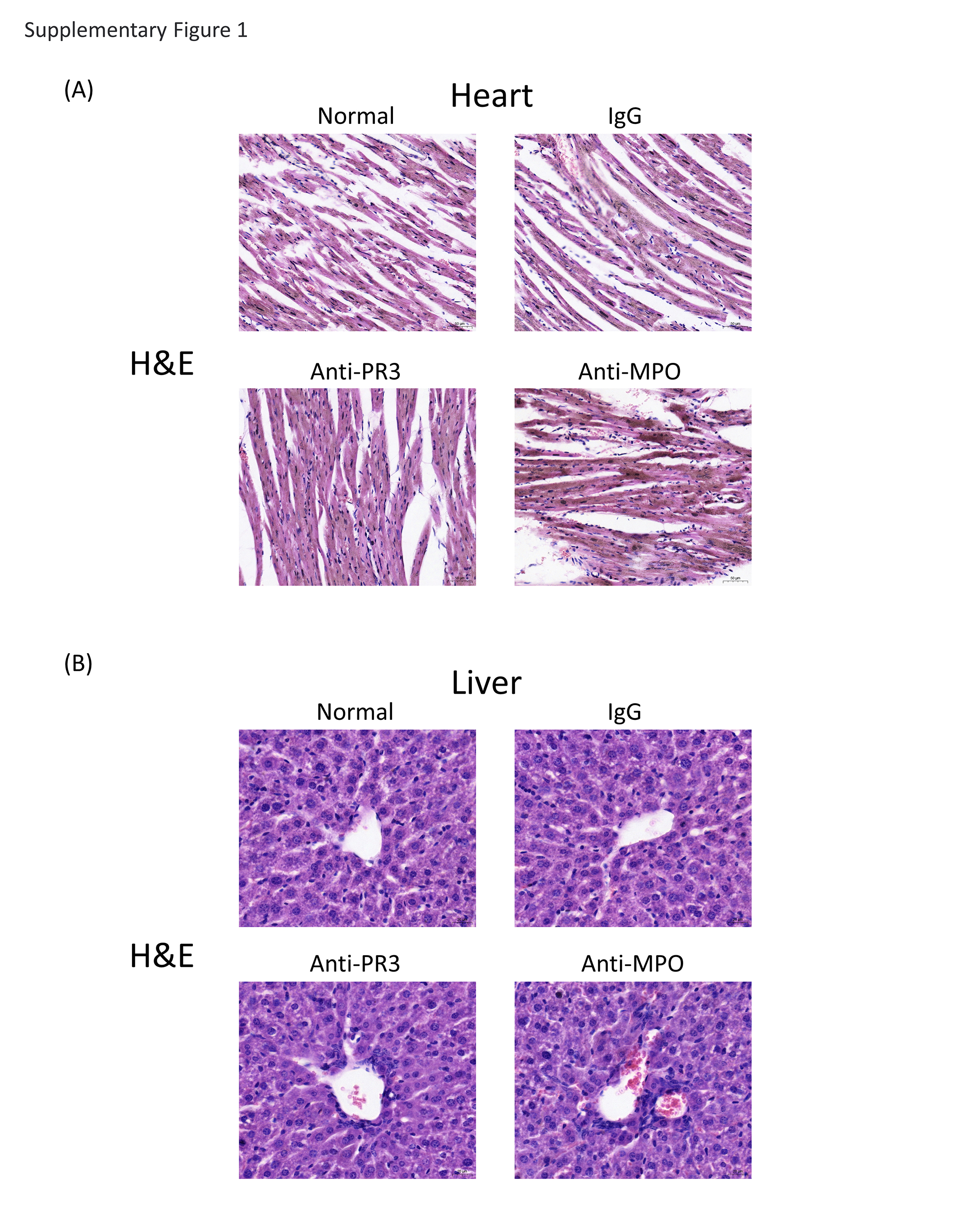

Supplement: Supplementary file 1 [file Image_1.jpeg]

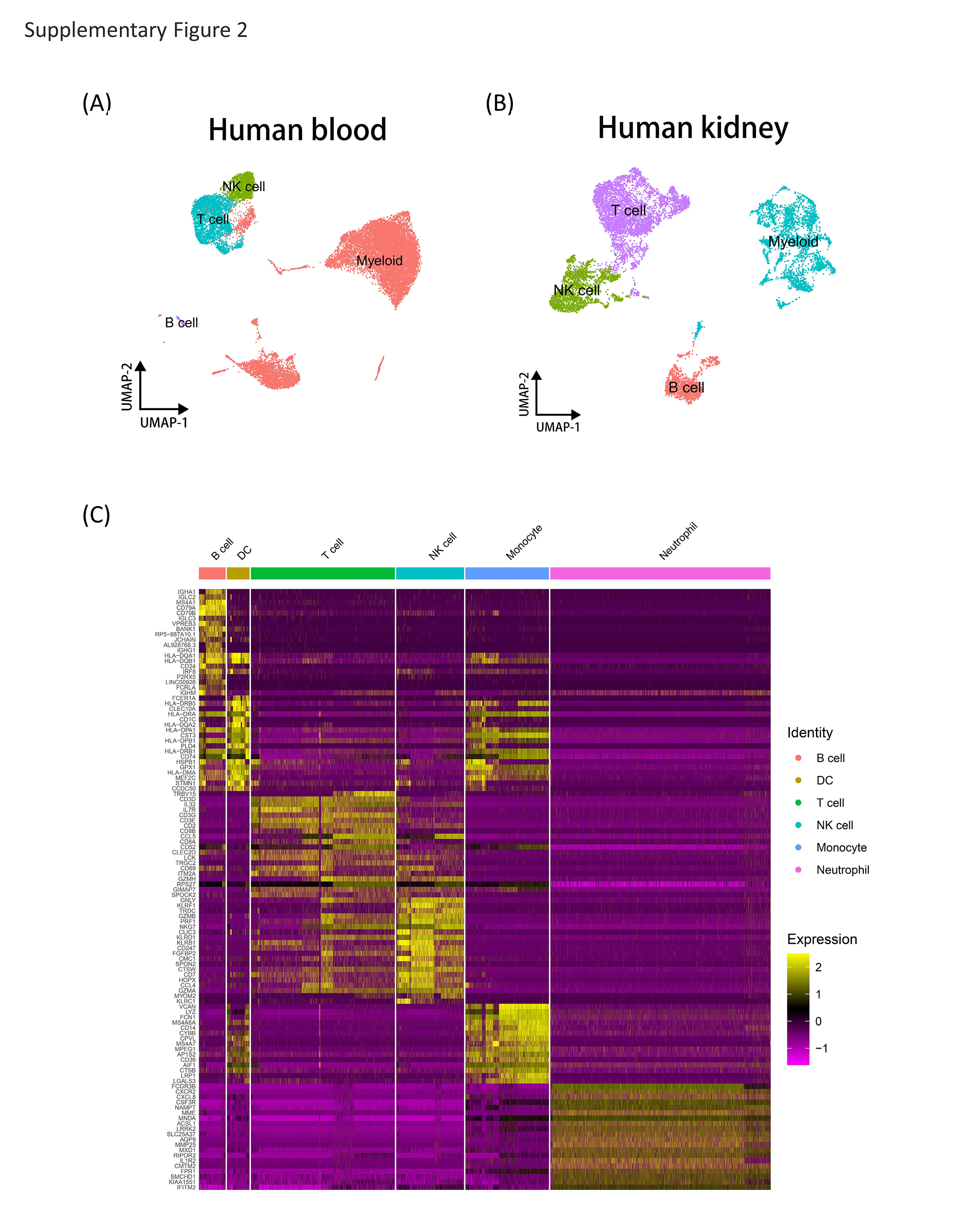

Supplement: Supplementary file 2 [file Image_2.jpeg]

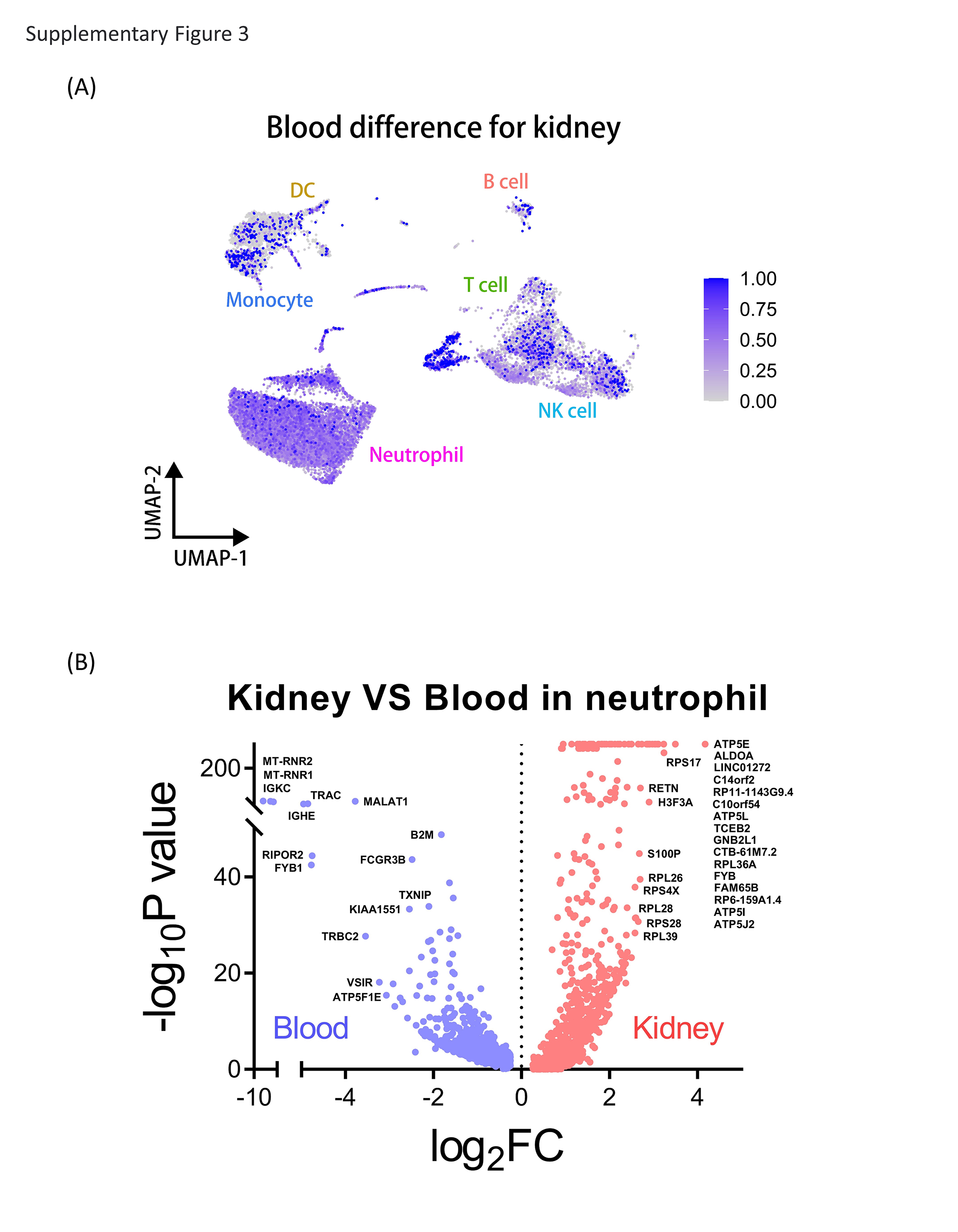

Supplement: Supplementary file 3 [file Image_3.jpeg]

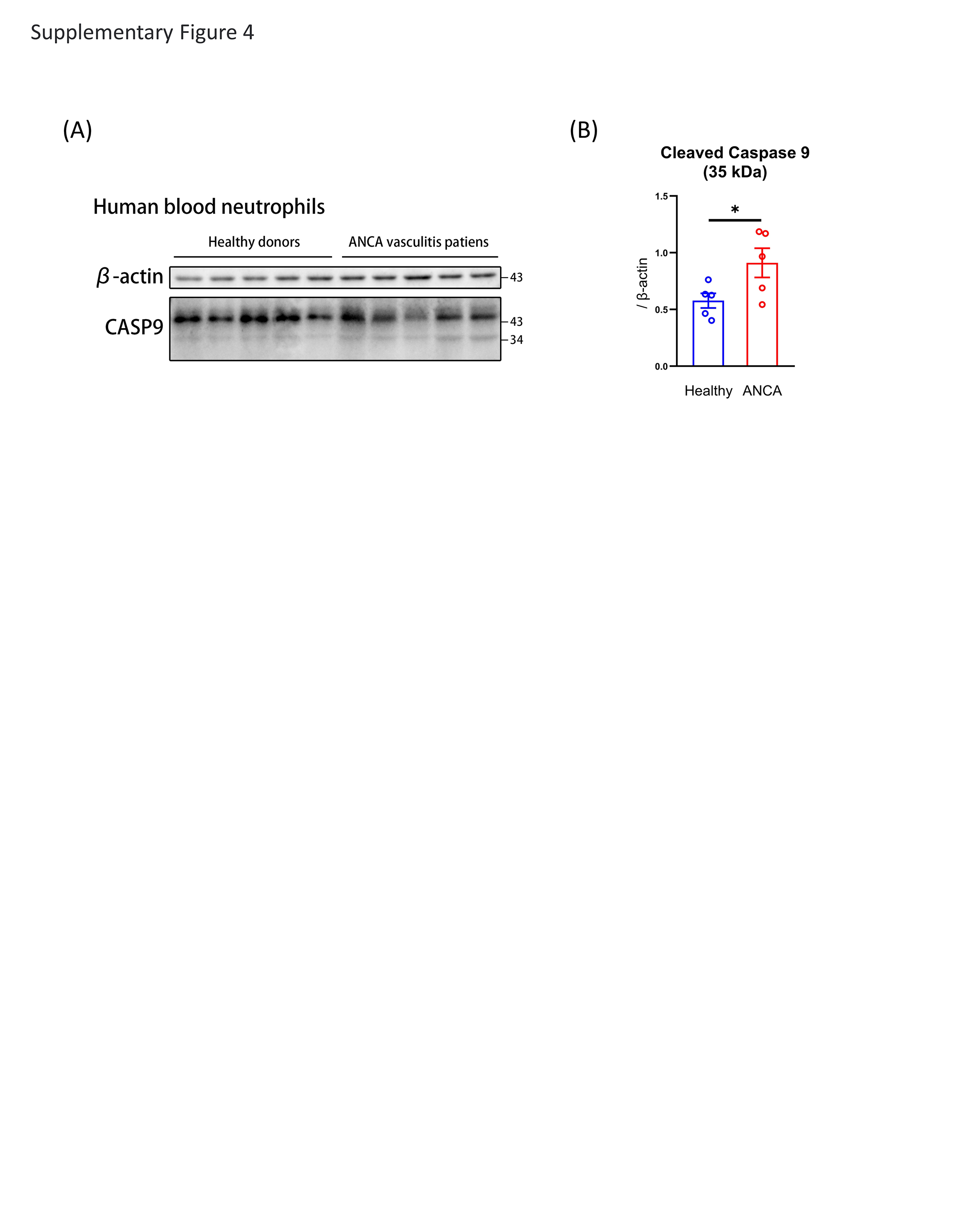

Supplement: Supplementary file 4 [file Image_4.jpeg]

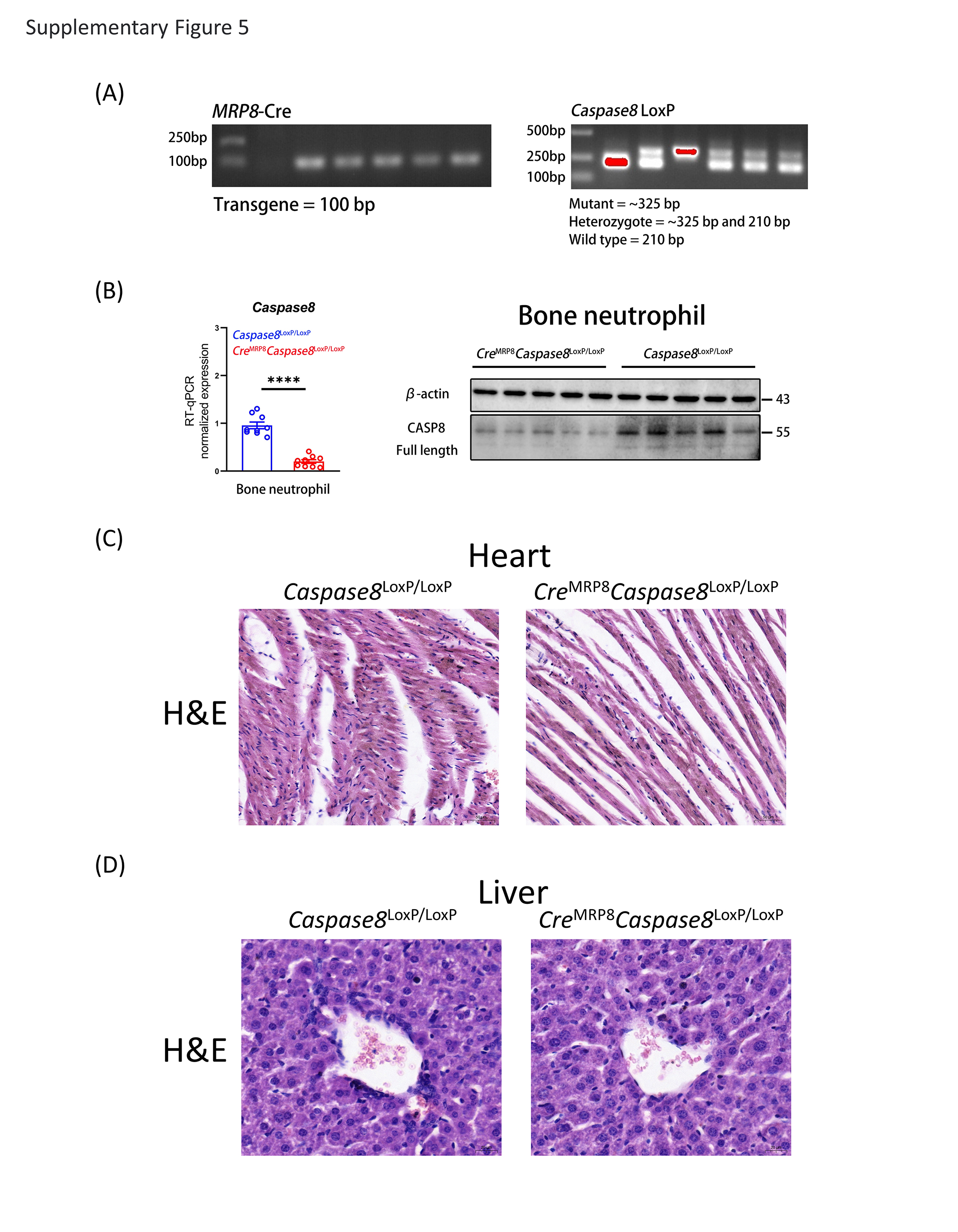

Supplement: Supplementary file 5 [file Image_5.jpeg]

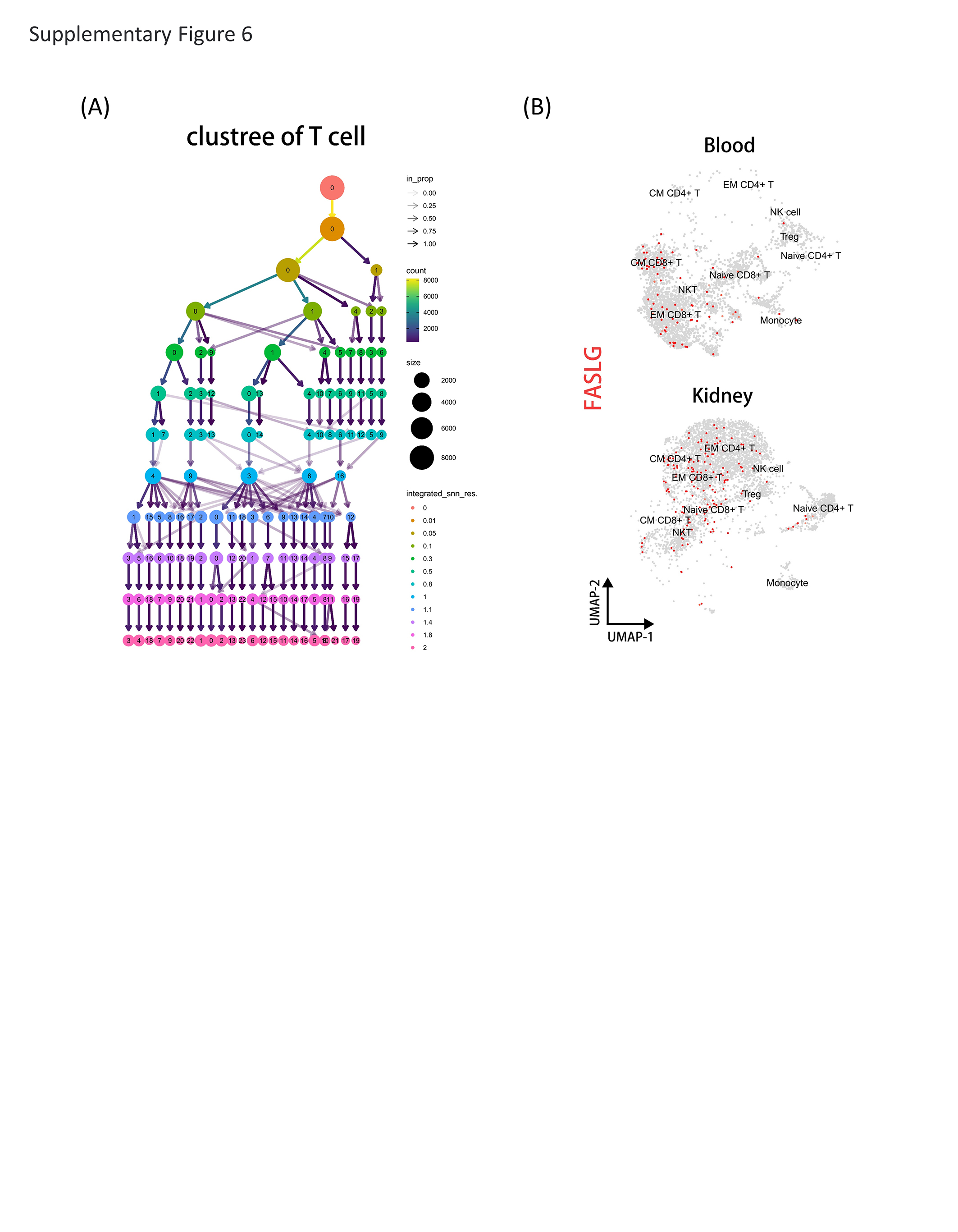

Supplement: Supplementary file 6 [file Image_6.jpeg]

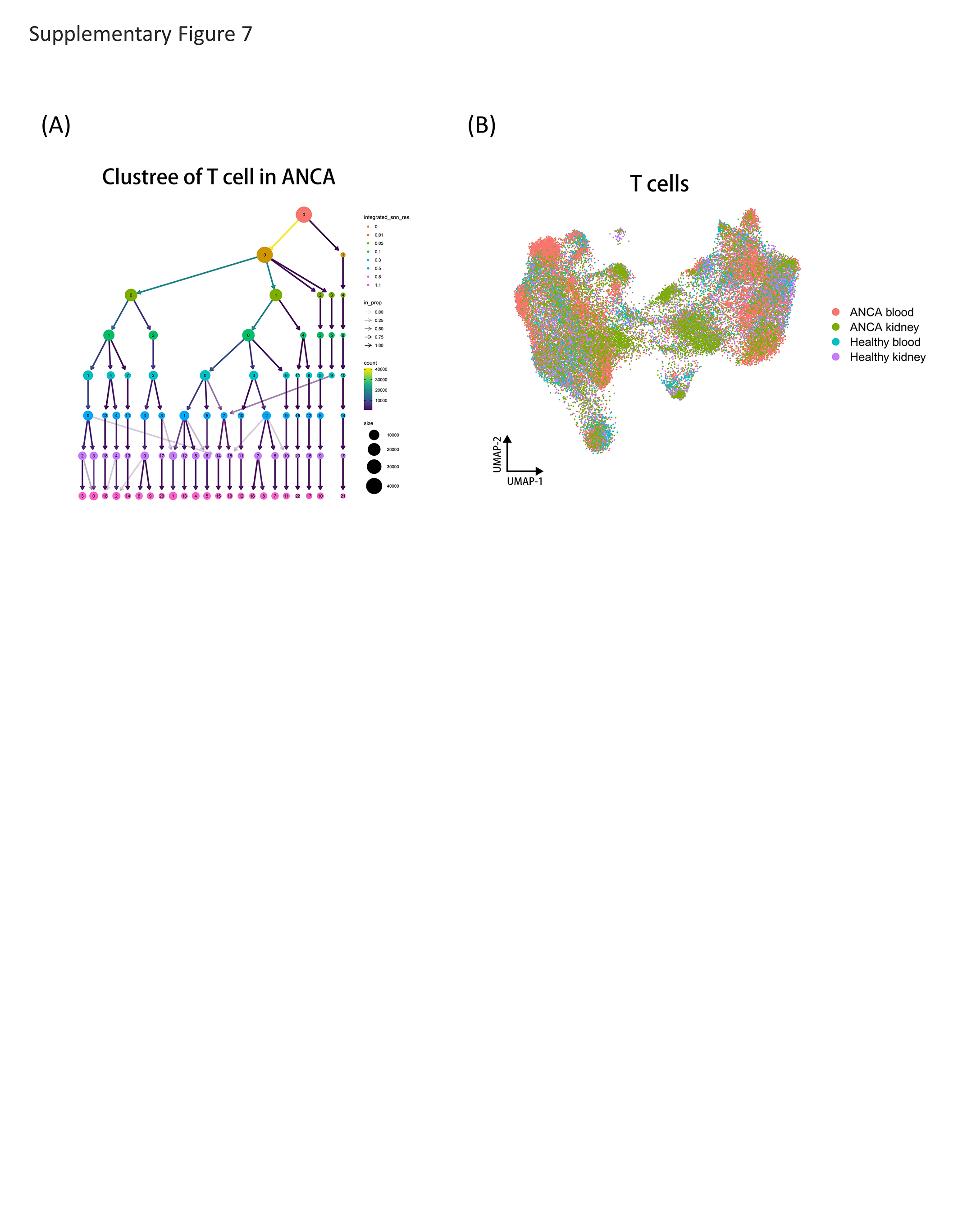

Supplement: Supplementary file 7 [file Image_7.jpeg]
